# A Linear Diffusion Model of Adsorption Kinetics at Fluid/Fluid Interfaces

**DOI:** 10.1007/s11743-016-1789-8

**Published:** 2016-01-22

**Authors:** Maciej Staszak

**Affiliations:** Institute of Chemical Technology and Engineering, Poznan University of Technology, Pl. Skłodowskiej-Curie 2, 60-965 Poznan, Poland

**Keywords:** Adsorption, Gibbs surface excess, Fick’s transient diffusion law, Sodium dodecyl sulfate

## Abstract

The paper presents a new model for kinetically controlled adsorption at the fluid/fluid interface. The main purpose of the presented approach is to relate easy to estimate bulk surfactant concentration with Gibbs surface excess. Two adsorption isotherms are involved in the new model development: Frumkin and Szyszkowski isotherms. Additionally the Johannsen time profile of concentration in the adsorption layer is assumed and estimated in the model derivation. The proposed approach assumes the near interface, adsorptive layer which is described based on Fick’s transient diffusion law. The solution to the model contains the estimation of effective diffusivities with adsorptive layer thickness as well. The experimental results of toluene/water + sodium dodecyl sulfate are presented and used for model verification.

## Introduction

The work presents a new kinetic approach to describe the adsorptive layer. The fundamental concept of the proposed mathematical model is to distinguish and analyze the space, located in the vicinity of the interface, where mass transfer is dominated by adsorptive forces. The description proposed is based on the Fick’s second diffusion law of molecular mass transport where the typical molecular diffusion coefficients are expressed as local effective diffusivities. Such an approach is typical for many systems in which the underlying transport mechanism is not yet clearly explained as for mass transfer in liquids or solids, in porous media or during some processes like extraction, drying, and many others.

A typical process in which the key phenomena takes place at the fluid interface is solvent extraction of metals using hydrophobic extractants. In order to estimate the rate of the process, dynamic surface tension experiments must be performed. This is achieved by forming a fresh interface, typically during the droplet creation, and analyzing the transient states of the surface tension. The kinetic interpretation divides the adsorption process into two parts. The first part concerns molecular diffusion from the bulk phase towards the interface. This process can be described by Fick’s laws or Maxwell–Stefan theory [1–5].

The second part is the mass transfer through the adsorptive sublayer located near the interface. In this space the main driving force is adsorption. Due to the nature of mass transport near the interface, the process must be described by a different mathematical approach. In this case the typical description includes the Fick’s law of mass transfer with the assumption that the diffusion coefficient is not molecular but attains some specific effective value. The values of the effective diffusion coefficients can differ greatly from the molecular ones.

Finally the adsorption itself is the mass exchange between interface and the sublayer space. The rate of the process can be thus limited by both phenomena, diffusion from the bulk to the sublayer and adsorption at the interface, or in the case of comparable rates, a diffusion-adsorptive process. In general, the adsorption step is faster than the diffusion so the kinetic description is typically limited to the diffusion process. But in the case of surfactants with a complicated structure, the diffusion process is not the only transport mechanism [6]. The limitations due to the geometric nature of the surfactant molecules or surfactant molecule arrangement at the interface can have a large impact on the adsorption rate.

The dynamics of the adsorption is described by three component fluxes given in Eq. 1:

1 $$\frac{{{\text{d}}\Gamma _{i} }}{{{\text{d}}t}} = J_{i}^{\text{ads}} - J_{i}^{\text{des}} + J_{i}^{\text{int}}$$

where *J*^ads^ is the adsorptive flux, *J*^des^ is the desorptive flux and *J*^int^ is the source flux. The internal source flux results from the reorganization of adsorbed molecules at the interface. In the case where the total surface excess does not uniquely explain the state of the interface, two characteristic states are introduced into the process description [7, 8]. These two states are characterized by different localization and distribution of adsorbed molecules. The variations of molecular orientation due to rotational and conformational changes results in changes in the surface density. In this case, the two states are characterized by two different surface excess values $\Gamma _{1}$, $\Gamma _{2}$ and different partial molar surfaces *ω*_1_ and *ω*_2_. The alteration and transition from one state to the other state is described by the so called two-state model. The resultant internal flux is proportional to the rate of reorientation described by the rate constant *k*_12_ and is given by Eq. 2:

2 $$J_{i}^{\text{int}} = k_{12} \left( {\left( {\frac{{\omega_{1} }}{{\omega_{2} }}} \right)^{\alpha } \left( {1 - \omega_{\text{mean}}\Gamma } \right)^{{\frac{{\omega_{1} - \omega_{2} }}{{\omega_{\text{mean}} }}}}\Gamma _{2} -\Gamma _{1} } \right)$$

The diffusion limited adsorption processes are described by several mathematical models, based on the Ward and Tordai approach [9]. They assumed the case where the interface is at equilibrium and compared the diffusional transport in the adsorptive sublayer to the bulk phase mass transport. They assumed that the only change in surface excess results from the diffusional surfactant mass flux *J*^dif^ given by Eq. 3.

3 $$\frac{{{\text{d}}\Gamma _{i} }}{{{\text{d}}t}} = J_{i}^{\text{dif}}$$

The description of the mass flux in the sublayer is based on Fick’s law where the molecular diffusion coefficient becomes effective. The effective diffusion coefficient *D*_ef_ takes into account the fact that the concentration gradient is not the primary driving force in the sublayer space. Consequently it is valid only in the vicinity of the interface and attains a different value than that for the bulk phase. Applying Fick’s second law to Eq. 3, the boundary condition at the interface expressed by the surface concentration as a function of time *c*_s_(*τ*) and the initial concentration *c*_0_ in the sublayer, gives Eq. 4.

4 $$\Gamma \left( t \right) = \sqrt {\frac{{D_{\text{ef}} }}{\pi }} \left[ {2c_{0} \sqrt t - \mathop \smallint \limits_{0}^{t} \frac{{c_{\text{s}} \left( \tau \right)}}{{\sqrt {t - \tau } }}d\tau } \right]$$

The choice of isotherm that relates the surface excess with the bulk phase concentration is important. Equation 4 demands specification of the time evolution of concentration at the interface given by the *c*_s_(*τ*) function in order to solve it analytically. There exist several simplified approaches, e.g., Fainermann [10] proposed the so called long and short time approximations which are widely used in the literature to give estimations of the diffusion coefficient in the sublayer. Direct estimation of diffusion coefficients using the Maxwell–Stefan approach is also presented by other works [11]. On the other hand, the kinetically controlled adsorption process kinetics is described by the rate constants *k*_ads_/*k*_des_ dependent on the temperature according to the Arrhenius–Eyring law [12]. The kinetics can be expressed by the typical Langmuir–Hinshelwood rate equation formulated in terms of surface excess Γ, its value at the saturated interface Γ_∞_ and the bulk concentration *c* given by Eq. 5.

5 $$\frac{{{\text{d}}\Gamma }}{{{\text{d}}t}} = k_{\text{ads}} c\left( {1 - \frac{\Gamma }{{\Gamma _{\infty } }}} \right) - k_{\text{des}}\Gamma .$$

Several other mixed-kinetic models are proposed where the process is limited by both the kinetics of adsorption and surfactant diffusion [13–23].

### Model Formulation

The space where the adsorptive forces tend to dominate is considered as a layer located near the interface and is assumed to play a fundamental role in the adsorption process. It is typical during fluid flow to consider two separate regions which differs by a specified property or set of properties. The Prandtl concept [24] of boundary layer is adapted in this work to the situation of adsorption sublayer near the interface. The bulk phase corresponds to the majority of the volume of the fluid. The boundary fluid layer is considered to have different properties than the bulk phase. In typical fluid flow analysis the properties can be chosen differently, e.g., velocity, heat transfer, concentration, mass flow, as well as several other parameters. Consequently the size of the layer depends on the specified type—thus the fluid surface can have multiple types of such layers defined differently at the same time [25]. The model presented in this work assumes the adsorptive layer is defined in terms of a space where adsorptive effects dominate, which is analogous to the aforementioned boundary layer concept. The model equations proposed are valid in the sublayer location and can be used to estimate the size of the layer. Real systems do not exhibit any sharp boundary between bulk and subsurface region, so in fact the size calculated from the model plays an estimative role and shows the order of magnitude of the adsorptive layer thickness.

### Model Derivation

**Fig. 1:**
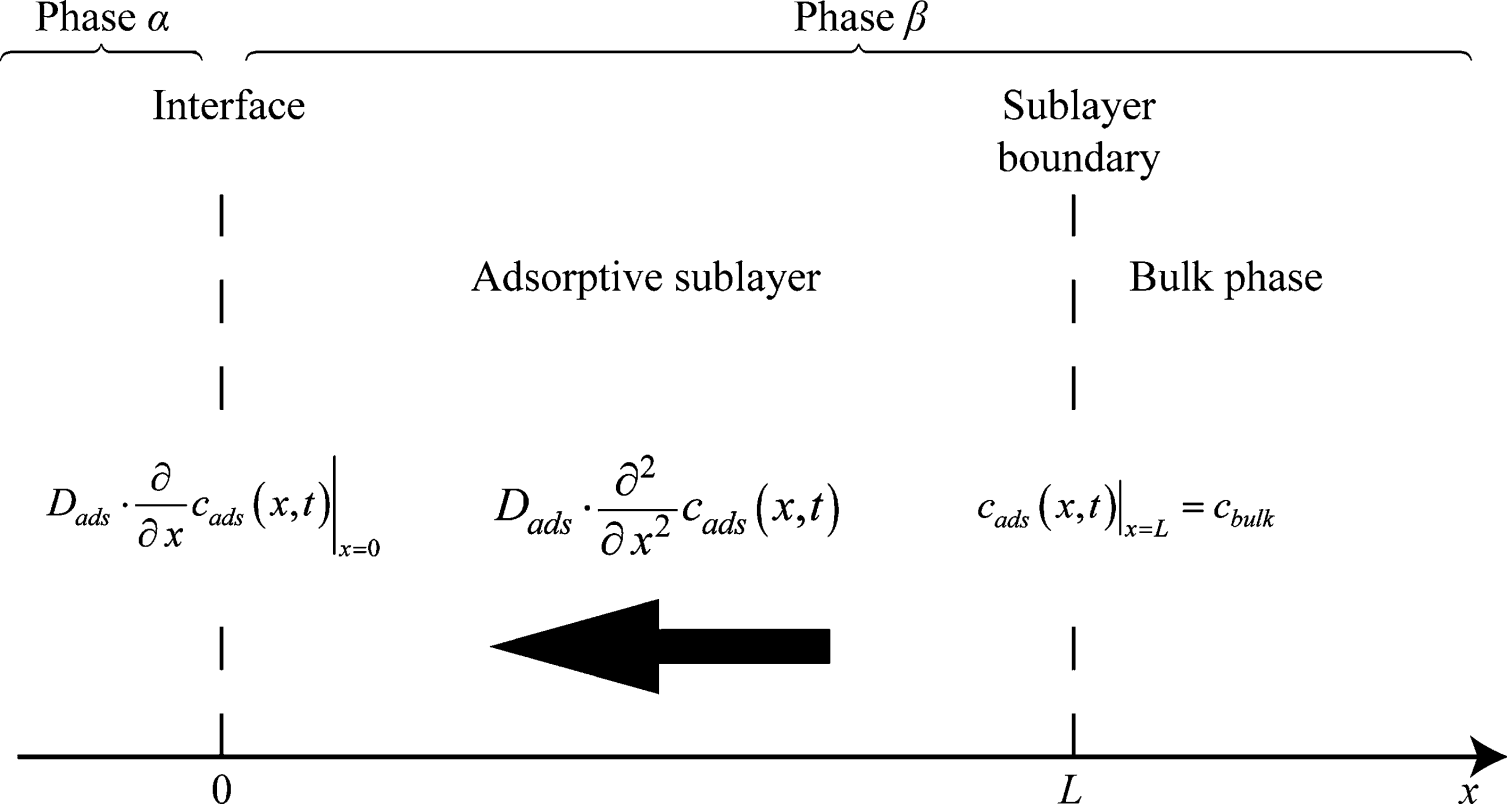
Adsorptive boundary layer and designation used in the derivation

Fick’s second law given by Eq. 6

6 $$D_{\text{ads}} \cdot \frac{{\partial^{2} }}{{\partial x^{2} }}c\left( {x,t} \right) = \frac{\partial }{\partial t}c\left( {x,t} \right)$$

is solved assuming the mass transfer in the adsorptive boundary layer is described by the effective diffusivity *D*_ads_. Because the layer is dominated by adsorptive forces, the classical correlations for bulk liquid phase diffusion coefficients are not suitable. In this case the diffusion coefficient becomes an effective diffusion coefficient for adsorption in the sublayer which holds the nature of the media, namely molecular diffusion with adsorptive driving forces. The concentration *c*(*x*, *t*) is the surfactant concentration in the sublayer at position *x* in process time *t*. Figure 1 presents the description of the model derivation, where the Gibbs assumption for zero interface thickness is applied.

The origin of the coordination system is fixed at the interface. The following boundary conditions are applied to the equation:

At the boundary of the adsorptive layer (*x* = *L*) it is assumed constant bulk concentration *c*_bulk_, by which the total bulk concentration is introduced for use in further modeling derivations.

7 $$\left. {c\left( {x,t} \right) = c_{\text{bulk}} } \right|_{x = L}$$

At the interface (*x* = 0) it is assumed that the total mass flux influences the Gibbs surface excess. Because Gibbs surface excess Γ is defined only at the interface location it is represented by an ordinary differential equation dependent only on *t*.

8 $$\left. {D_{\text{ads}} \cdot \frac{\partial }{\partial x}c\left( {x,t} \right) = \frac{\text{d}}{{{\text{d}}t}}\Gamma \left( t \right)} \right|_{x = 0}$$

The initial condition for the boundary layer can be proposed in several ways. One possibility is to set the adsorptive layer specie concentration *c*(*x*, 0) equal to *c*_bulk_ which is the situation in the moment of contact of the phases.

To find the general solution to Eq. 6, the separation of variables [26] method is applied. By assuming that the solution can be presented as the product of two functions:

9 $$c\left( {x,t} \right) = T\left( t \right)X\left( x \right)$$

and substituting (9) into (6), yields Eq. 10.

10 $$\frac{1}{T\left( t \right)D}\frac{\text{d}}{{{\text{d}}t}}T\left( t \right) = \frac{1}{X\left( x \right)}\frac{{{\text{d}}^{2} }}{{{\text{d}}x^{2} }}X\left( x \right).$$

The left and right-hand sides of the equation are set equal to a specified parameter, which must be arbitrarily selected according to the required form of solution. Because diffusive mass transport of surfactant in the adsorption layer produces an exponential rise of concentration along its width *x*, the constant is chosen to give an exponential form.

11 $$\left\{ \begin{aligned} \frac{1}{{T\left( t \right)D_{\text{ads}} }}\frac{\text{d}}{{{\text{d}}t}}T\left( t \right) = \omega , \\ \frac{1}{X\left( x \right)}\frac{{{\text{d}}^{2} }}{{{\text{d}}x^{2} }}X\left( x \right) = \omega . \\ \end{aligned} \right.$$

The dimension of the selected constant *ω* is the reciprocal of area m^−2^. It should be mentioned that the possibility exists to choose other constants which gives a trigonometric form of integration result. Equation 11 are independently solved to give solutions which have an exponential form:

12 $$\left\{ \begin{array}{ll} T\left( t \right) = {\text{e}}^{{\omega D_{\text{ads}} t}} , \hfill \\ X\left( x \right) = A{\text{e}}^{\sqrt \omega x} + B{\text{e}}^{ - \sqrt \omega x} . \hfill \\ \end{array} \right.$$

where the two constants *A* and *B* that appear in the solution (12) depend on the chosen boundary conditions. Using the assumption in Eq. 9, the general solution to the diffusion equation is obtained. The general solution to the Eq. (6) contains three parameters that must be specified according to the nature of the adsorption layer.

13 $${\text{c}}\left( {x,t} \right){\text{ = e}}^{{\omega D_{\text{ads}} t}} \left( {A{\text{e}}^{\sqrt \omega x} + B{\text{e}}^{ - \sqrt \omega x} } \right)$$

The parameters are to be formulated in terms of boundary conditions (7) and (8) to obtain a particular solution for the case analyzed. Applying the boundary condition at *x* = *L* to the general solution (13) gives:

14 $$c_{\text{bulk}} = {\text{e}}^{{\omega D_{\text{ads}} t}} \left( {A{\text{e}}^{\sqrt \omega L} + B{\text{e}}^{ - \sqrt \omega L} } \right).$$

By isolating constant *A*,

15 $$A = \left( {\frac{{c_{\text{bulk}} }}{{{\text{e}}^{{\omega D_{\text{ads}} }} }} - B{\text{e}}^{ - \sqrt \omega L} } \right){\text{e}}^{ - \sqrt \omega L}$$

and applying it to general solution (13) one of the integration constants is removed:

16 $$c\left( {x,t} \right) = \left( { - \left( {{\text{e}}^{{ - \sqrt \omega \left( {2L - x} \right)}} - {\text{e}}^{ - \sqrt \omega x} } \right)B + {\text{e}}^{{ - \omega D_{\text{ads}} t - \sqrt \omega L + \sqrt \omega x}} c_{\text{bulk}} } \right){\text{e}}^{{\omega D_{\text{ads}} t}}$$

Consequently applying the boundary condition (8) at the interface *x* = 0 to (16) gives:

17 $${\text{e}}^{{\omega D_{\text{ads}} t}} \left( { - \left( { - c_{\text{bulk}} + B{\text{e}}^{{ - \sqrt \omega L + \omega D_{\text{ads}} t}} } \right){\text{e}}^{{ - \omega D_{\text{ads}} t - \sqrt \omega L}} \sqrt \omega - B\sqrt \omega } \right) = \frac{{\frac{\text{d}}{{{\text{d}}t}}\Gamma \left( t \right)}}{{D_{\text{ads}} }}$$

Isolating *B* allows us to reduce the number of constants existing in the general solution (13):

18 $$B = \frac{{\left( { - \left( {\frac{\text{d}}{{{\text{d}}t}}\Gamma \left( t \right)} \right) + {\text{e}}^{ - \sqrt \omega L} \sqrt \omega c_{\text{bulk}} D_{\text{ads}} } \right){\text{e}}^{{ - \omega D_{\text{ads}} t}} }}{{D_{\text{ads}} \sqrt \omega \left( {{\text{e}}^{ - 2\sqrt \omega L} + 1} \right)}}$$

Removing the constant *B* and isolating the differential dΓ/d*t* allows us to express the general change of the surface excess Γ due to adsorptive layer surfactant concentration transport based on the obtained particular solution:

19 $$c\left( {x,t} \right) = - \frac{{ - {\text{e}}^{{\left( {x - L} \right)\sqrt \omega }} c_{\text{bulk}} D_{\text{ads}} \sqrt \omega + \frac{\text{d}}{{{\text{d}}t}}\Gamma \left( t \right)\left( {{\text{e}}^{ - \sqrt \omega x} - {\text{e}}^{{\sqrt \omega \left( {x - 2L} \right)}} } \right) - {\text{e}}^{{ - \sqrt \omega \left( {x + L} \right)}} \sqrt \omega c_{\text{bulk}} D_{\text{ads}} }}{{D_{\text{ads}} \sqrt \omega \left( {{\text{e}}^{ - 2\sqrt \omega L} + 1} \right)}}$$

The result above is rearranged to isolate the change of Gibbs surface excess in time, which yields:

20 $$\frac{\text{d}}{{{\text{d}}t}}\Gamma \left( {t,c_{\text{bulk}} } \right) = \frac{{\sqrt \omega D_{\text{ads}} \left( {c\left( {x,t} \right){\text{e}}^{ - 2\sqrt \omega L} + c\left( {x,t} \right) - {\text{e}}^{{ - \left( {L - x} \right)\sqrt \omega }} c_{\text{bulk}} - {\text{e}}^{{ - \sqrt \omega \left( {x + L} \right)}} c_{\text{bulk}} } \right)}}{{{\text{e}}^{{ - \sqrt \omega \left( {2L - x} \right)}} - {\text{e}}^{ - \sqrt\omega x} }}$$

Equation (20) represents the differential equation, with formal assignment of Gibbs excess dependence on the bulk concentration *c*_bulk_. The term *c*(*x*,*t*) represents the general dependence of concentration on time *t* and (only formally specified) on distance *x* at the surface. To solve Eq. 20 by analytical means, some specific formulation must be chosen. For this purpose the Johannsen [27] surfactant concentration time profile in the adsorptive layer is applied:

21 $$c\left( {x,t} \right) = c_{\text{bulk}} \left( {1 - {\text{e}}^{ - at} + {\text{e}}^{ - bt} } \right)$$

in which the *a* and *b* are constants specific to the kinetics of the analyzed system. The Johannsen equation is applied for *x* = 0. Reformulated this way Eq. 20 becomes,

22 $$\frac{\text{d}}{{{\text{d}}t}}\Gamma \left( {t,c_{\text{bulk}} } \right) = \frac{{\left( \begin{array}{l} {\text{e}}^{ - 2\sqrt \omega L} + 1 - {\text{e}}^{ - at - 2\sqrt \omega L} - {\text{e}}^{ - at} + {\text{e}}^{{ - bt - 2\sqrt {\text{v}} L}} \hfill \\ \; \cdots + {\text{e}}^{ - bt} - {\text{e}}^{{ - \left( {L - x} \right)\sqrt \omega }} - {\text{e}}^{{ - \sqrt \omega \left( {x + L} \right)}} \hfill \\ \end{array} \right)c_{\text{bulk}} D_{\text{ads}} \sqrt \omega }}{{{\text{e}}^{{ - \sqrt \omega \left( {2L - x} \right)}} - {\text{e}}^{ - \sqrt \omega x} }}$$

which is a description of the Gibbs surface excess change in time according to the boundary conditions and the assumption of a Johannsen concentration time profile on the interface. Equation (22) formulates the initial value problem and for this the initial condition must be applied to obtain a particular solution. First, integrating the equation one obtains the general solution for surface excess Γ evolution:

23 $$\Gamma \left( {t,c_{\text{bulk}} } \right) = \frac{{c_{\text{bulk}} D_{\text{ads}} \sqrt \omega \left( \begin{array}{l} {\text{e}}^{ - 2\sqrt \omega L} t + t + \frac{{{\text{e}}^{ - at - 2\sqrt \omega L} }}{a} + \frac{{{\text{e}}^{ - at} }}{a} - \frac{{{\text{e}}^{ - bt - 2\sqrt \omega L} }}{b} \hfill \\ \; \cdots - \frac{{{\text{e}}^{ - bt} }}{b} - {\text{e}}^{{ - \left( {L - x} \right)\sqrt \omega }} t - {\text{e}}^{{ - \sqrt \omega \left( {x + L} \right)}} t \hfill \\ \end{array} \right)}}{{{\text{e}}^{{ - \sqrt \omega \left( {2L - x} \right)}} - {\text{e}}^{ - \sqrt \omega x} }} + C$$

At this point the initial state of the surface excess is assumed to be described by the function depending on bulk concentration Γ_init_(*c*_bulk_). It refers to the typical situation when some initial state exists in the system which is at equilibrium with the bulk concentration and at specified initial moment when this bulk concentration is altered. This is mathematically more complex then applying trivial zero initial Gibbs surface excess, but allows us to track the dynamics of the surface excess in a more general way. Applying initial condition Γ_init_(*c*_bulk_) at *t* = 0 gives:

24 $$\begin{aligned}\Gamma (t,c_{\text{bulk}} ) = \frac{{c_{\text{bulk}} D_{\text{ads}} \sqrt \omega \left( \begin{array}{l} - {\text{e}}^{ - 2\sqrt \omega L} t - t - \frac{{{\text{e}}^{ - at - 2\sqrt \omega L} }}{a} - \frac{{{\text{e}}^{ - at} }}{a} + \frac{{{\text{e}}^{ - bt - 2\sqrt \omega L} }}{b} \hfill \\ \; \cdots + \frac{{{\text{e}}^{ - bt} }}{b} + {\text{e}}^{{ - \left( {L - x} \right)\sqrt \omega }} t + {\text{e}}^{{ - \sqrt \omega \left( {x + L} \right)}} t \hfill \\ \end{array} \right)}}{{ - {\text{e}}^{{ - \sqrt \omega \left( {2L - x} \right)}} + {\text{e}}^{ - \sqrt \omega x} }} \\ \quad \; \cdots - \frac{{c_{\text{bulk}} D_{\text{ads}} \sqrt \omega \left( { - \frac{{{\text{e}}^{ - 2\sqrt \omega L} }}{a} - \frac{1}{a} + \frac{{{\text{e}}^{ - 2\sqrt \omega L} }}{b} + \frac{1}{b}} \right)}}{{ - {\text{e}}^{{ - \sqrt \omega \left( {2L - x} \right)}} + {\text{e}}^{ - \sqrt \omega x} }} +\Gamma _{\text{init}} \left( {c_{\text{bulk}} } \right) \\ \end{aligned}$$

The right-hand side of Eq. 24 is defined in terms of the spatial variable *x* which in fact has no physical meaning except at *x* = 0 (interface location). Applying the interface condition of *x* = 0 simplifies (24) to give:

25 $$\begin{aligned}\Gamma \left( t \right) = \frac{{c_{\text{bulk}} D_{\text{ads}} \sqrt \omega \left( \begin{array}{l} - {\text{e}}^{ - 2\sqrt \omega L} t - t - \frac{{{\text{e}}^{ - at - 2\sqrt \omega L} }}{a} - \frac{{{\text{e}}^{ - at} }}{a} + \frac{{{\text{e}}^{ - bt - 2\sqrt \omega L} }}{b} \hfill \\ \; \cdots + \frac{{{\text{e}}^{ - bt} }}{b} + 2{\text{e}}^{ - \sqrt \omega L} t \hfill \\ \end{array} \right)}}{{ - {\text{e}}^{ - 2\sqrt \omega L} + 1}} \\ \quad \cdots - \frac{{c_{\text{bulk}} D_{\text{ads}} \sqrt \omega \left( { - \frac{{{\text{e}}^{ - 2\sqrt \omega L} }}{a} - \frac{1}{a} + \frac{{{\text{e}}^{ - 2\sqrt \omega L} }}{b} + \frac{1}{b}} \right)}}{{ - {\text{e}}^{ - 2\sqrt \omega L} + 1}} +\Gamma _{\text{init}} \left( {c_{\text{bulk}} } \right) \\ \end{aligned}$$

Equation 25 contains several variables which must be estimated in order to prove its usefulness for describing the interface adsorption kinetics. The most fundamental are effective diffusion coefficient *D*_ads_ and adsorptive layer thickness *L*. The constant *ω*, which results from integration of diffusion Eq. (6), is constrained to positive values only. The constants *a* and *b* are introduced by Johannsen equilibrium profile assumption (in original text *β*_*1*_ and *β*_*2*_) and refer to the adsorptive and desorptive fluxes when the interface condition shifts towards the equilibrium state.

### Initial Condition

The description of initial condition Γ_init_(*c*_bulk_) at *t* = 0 to Eq. 25 can be done based on the assumption that, at the initial point of the experiment, the system maintains the equilibrium state. The initial equilibrium state, which correspond to the initial bulk concentration (*c*_bulk_ at *t* = 0) used for sample preparation, is then disturbed by the change of the bulk surfactant concentration. There also exists the possibility to apply non-equilibrium initial condition, e.g., zero value of Gibbs surface excess which leads to a simpler derivation. At this point it is assumed some local equilibrium state exists and is described by typical isotherms used to describe liquid/liquid adsorption.

### Szyszkowski Isotherm

The well-known Szyszkowski isotherm which was derived from the Gibbs and Langmuir isotherms, assumes ideal behavior of the bulk surfactant component. That leads to a condition of dilute solution of the surface active specie in the bulk phase. The classical formulation [28] of the Szyszkowski isotherm in terms of the maximum surface excess Γ_∞_ and adsorption equilibrium constant *K*_L_ from the Langmuir isotherm reads:

26 $$\gamma_{0} - \gamma = nRT\Gamma _{\infty } \ln \left( {1 + K_{\text{L}} c_{\text{bulk}} } \right).$$

The empirical formulation of the Szyszkowski equation [29, 30] relates surface tension *γ* to the bulk concentration *c*_bulk_ by the use of *A*_Sz_ and *B*_Sz_ Szyszkowski constants:

27 $$\gamma = \gamma_{0} \left( {1 - B_{\text{Sz}} \ln \left( {\frac{{c_{\text{bulk}} }}{{A_{\text{Sz}} }} + 1} \right)} \right).$$

The Gibbs surface excess in terms of Szyszkowski constants is then presented by the relation (28):

28 $$\Gamma = \frac{{B_{\text{Sz}} g_{0} c_{\text{bulk}} }}{{RT\left( {c_{\text{bulk}} + A_{\text{Sz}} } \right)}}.$$

Applying (28) as the initial condition to (25) for Γ_init_ gives the formulation for the time evolution of surface excess based on the Szyszkowski isotherm:

29 $$\begin{aligned}\Gamma \left( t \right) = \frac{{c_{\text{bulk}} D_{\text{ads}} \sqrt \omega \left( { - {\text{e}}^{ - 2\sqrt \omega L} t - t - \frac{{{\text{e}}^{ - at - 2\sqrt \omega L} }}{a} - \frac{{{\text{e}}^{ - at} }}{a} + \frac{{{\text{e}}^{ - bt - 2\sqrt \omega L} }}{b} + \frac{{{\text{e}}^{ - bt} }}{b} + 2{\text{e}}^{ - \sqrt \omega L} t} \right)}}{{ - {\text{e}}^{ - 2\sqrt \omega L} + 1}} \\ \quad \cdots + \frac{{B_{Sz} g_{0} c_{\text{bulk}} }}{{RT\left( {c_{\text{bulk}} + A_{\text{Sz}} } \right)}} - \frac{{c_{\text{bulk}} D_{\text{ads}} \sqrt \omega \left( { - \frac{{{\text{e}}^{ - 2\sqrt \omega L} }}{a} - \frac{1}{a} + \frac{{{\text{e}}^{ - 2\sqrt \omega L} }}{b} + \frac{1}{b}} \right)}}{{ - {\text{e}}^{ - 2\sqrt \omega L} + 1}} \\ \end{aligned}$$

### Frumkin Isotherm

The Langmuir isotherm is transformed to the Frumkin isotherm by considering the lateral interactions between adsorbed molecules. The additional interaction parameter *A*′ attains negative values for repulsion and positive for attraction forces. The formulation of the Szyszkowski–Langmuir equation [28] in the sense of surface excess reads:

30 $$\gamma_{0} - \gamma = - nRT\Gamma _{\infty } \ln \left( {1 - \frac{\Gamma }{{\Gamma _{\infty } }}} \right).$$

The Frumkin empirical equation relates surface tension to bulk concentration incorporating three constants *A*_Fr_, *B*_Fr_ and *A*′.

31 $$\gamma = \gamma_{0} \left( {1 - B_{\text{Fr}} \ln \left( {\frac{{c_{\text{bulk}} }}{{A_{\text{Fr}} }} + 1} \right) - \frac{{A^{\prime } c^{2} }}{{\left( {c_{\text{bulk}} + A_{\text{Fr}} } \right)^{2} }}} \right).$$

where:

32 $$A^{{\prime }} = - \frac{N\varphi }{2kT}.$$

in which *φ* is the energy of interaction between one pair of adsorbed molecules, and the term *Nφ* is the interaction of one molecule with its *N* nearest neighbors in the totally covered surface [31]. Applying (31) to the Gibbs isotherm gives:

33 $$\Gamma = - \frac{{c_{\text{bulk}} \gamma_{0} }}{RT}\left( { - \frac{{B_{\text{Fr}} }}{{A_{\text{Fr}} \left( {\frac{{c_{\text{bulk}} }}{{A_{\text{Fr}} }} + 1} \right)}} + \frac{{2A^{{\prime }} c_{\text{bulk}} A_{\text{Fr}} }}{{\left( {c_{\text{bulk}} + A_{\text{Fr}} } \right)^{3} }}} \right).$$

The above equations are given in a form [42] to enable convenient comparisons between parameters of both isotherms. For the model presented, Eq. 33 is applied as an initial condition Γ_init_ to (25) and formulates the time evolution in the sense of Frumkin isotherm allowing for an additional estimate of the interaction parameter *A*′.

34 $$\begin{aligned}\Gamma \left( t \right) & = \frac{{c_{\text{bulk}} D_{\text{ads}} \sqrt \omega \left( { - {\text{e}}^{ - 2\sqrt \omega L} t - t - \frac{{{\text{e}}^{ - at - 2\sqrt \omega L} }}{a} - \frac{{{\text{e}}^{ - at} }}{a} + \frac{{{\text{e}}^{ - bt - 2\sqrt \omega L} }}{b} + \frac{{{\text{e}}^{ - bt} }}{b} + 2{\text{e}}^{ - \sqrt \omega L} t} \right)}}{{ - {\text{e}}^{ - 2\sqrt \omega L} + 1}} \\ &\qquad \cdots + - \frac{{c_{\text{bulk}} \gamma_{0} }}{RT}\left( { - \frac{{B_{\text{Fr}} }}{{A_{\text{Fr}} \left( {\frac{{c_{\text{bulk}} }}{{A_{\text{Fr}} }} + 1} \right)}} + \frac{{2A^{{\prime }} c_{\text{bulk}} A_{\text{Fr}} }}{{\left( {c_{\text{bulk}} + A_{\text{Fr}} } \right)^{3} }}} \right) - \frac{{c_{\text{bulk}} {\text{D}}_{\text{ads}} \sqrt \omega \left( { - \frac{{{\text{e}}^{ - 2\sqrt \omega L} }}{a} - \frac{1}{a} + \frac{{{\text{e}}^{ - 2\sqrt \omega L} }}{b} + \frac{1}{b}} \right)}}{{ - {\text{e}}^{ - 2\sqrt \omega L} + 1}} \\ \end{aligned}$$

### Model Validity

The model derivation is a balance between its complexity and ability to obtain an analytical solution. It is evident that the model analytical formulation contains a time variable *t* as a free term which for large values of time will not render the equilibrium value of Γ. It is then necessary to examine the extent to which the model gives reasonable estimates. With the character of sigmoidal growth it is safe to assume that the deflection point of time evolution of excess is a safe location of the model application validity range. This can be done by equating the third derivative of the formulation for Gibbs surface excess to zero and finding limiting time *t*:

35 $$\frac{{{\text{d}}^{3}\Gamma }}{{{\text{d}}t^{3} }} = \frac{{D_{\text{ads}} \sqrt \omega c_{\text{bulk}} \left( { - a^{2} {\text{e}}^{ - at + 2\sqrt \omega L} - a^{2} {\text{e}}^{ - at} + b^{2} {\text{e}}^{ - bt + 2\sqrt \omega L} + b^{2} {\text{e}}^{ - bt} } \right)}}{{ - 1 + {\text{e}}^{2\sqrt \omega L} }} = 0$$

The solution to the above reads:

36 $$t_{\hbox{max} } = \frac{{\ln \left( {\frac{{a^{2} }}{{b^{2} }}} \right)}}{a - b}$$

The value of *t*_max_ is cautious and conservative which ensures that the model will not give a result exceeding Γ_∞_ and the value of *t*_max_ itself is of the order of the relaxation time of diffusion in the adsorptive sublayer of width *L*.

## Experimental Results

### Chemicals

Experimental surface tension data is needed to validate the proposed model. In this work, a water + sodium dodecyl sulfate (SDS)/toluene system was selected as a test of the proposed model. Sodium dodecyl sulfate was obtained from Sigma-Aldrich with a purity of 99 % and used without further purification. SDS is water soluble and does not transfer to organic phase. The additional reason to use SDS is fact that its surface properties are very well known thus it is a good test system for the presented model. All the surfactants solutions were prepared by using water from the PURELAB Classic, Elga with a resistivity of 18.2 MΩ cm. Toluene (pure p.a. from Avantor Performance Materials POCH™) was distilled before use. Samples were shaken for 4 h to assure the equilibrium state at the interface. The measurements were carried out at 25 °C.

### Estimation of Isotherms Parameters

The calculation of isotherms parameters is straightforward and is done using a least squares algorithm. The calculated isotherms’ parameters are needed for further calculation and for the comparison with the proposed model. The calculated data sets (Table 4) are in general accordance to other works [32, 33]. The goodness of fit, to be comparable between models with different number of parameters, is calculated as a square sum of errors divided by the model degree of freedom. The degree of freedom is the number of measurements minus the number of parameters estimated in the model. The values presented indicate good fit for both isotherms. The lower value of goodness of fit for the simpler Szyszkowski isotherm is related to the number of measurements compared to the number of estimated parameters.

### Modeling Procedure

The experimental data preparation is done by the following method. The two proposed model solutions (29) and (34) for the two isotherms are used to estimate the diffusion coefficient *D*_ads_ in adsorption layer, its size *L*, and parameters *a* and *b* from the Johannsen Eq. (21). Additionally the *ω* parameter is estimated along with the correction of the isotherm parameters. During experimental measurements by the tensometric techniques the surface tension is obtained in relation to the actual bulk surfactant concentration. If the measurement is done over a long time period then the resultant values are equilibrium surface tensions. In the case of short time period the measurements are understood as dynamic, dependent on process time because the equilibrium state is not established in such cases. Both types of measurements are needed to perform calculations using the proposed approach.

**Fig. 2:**
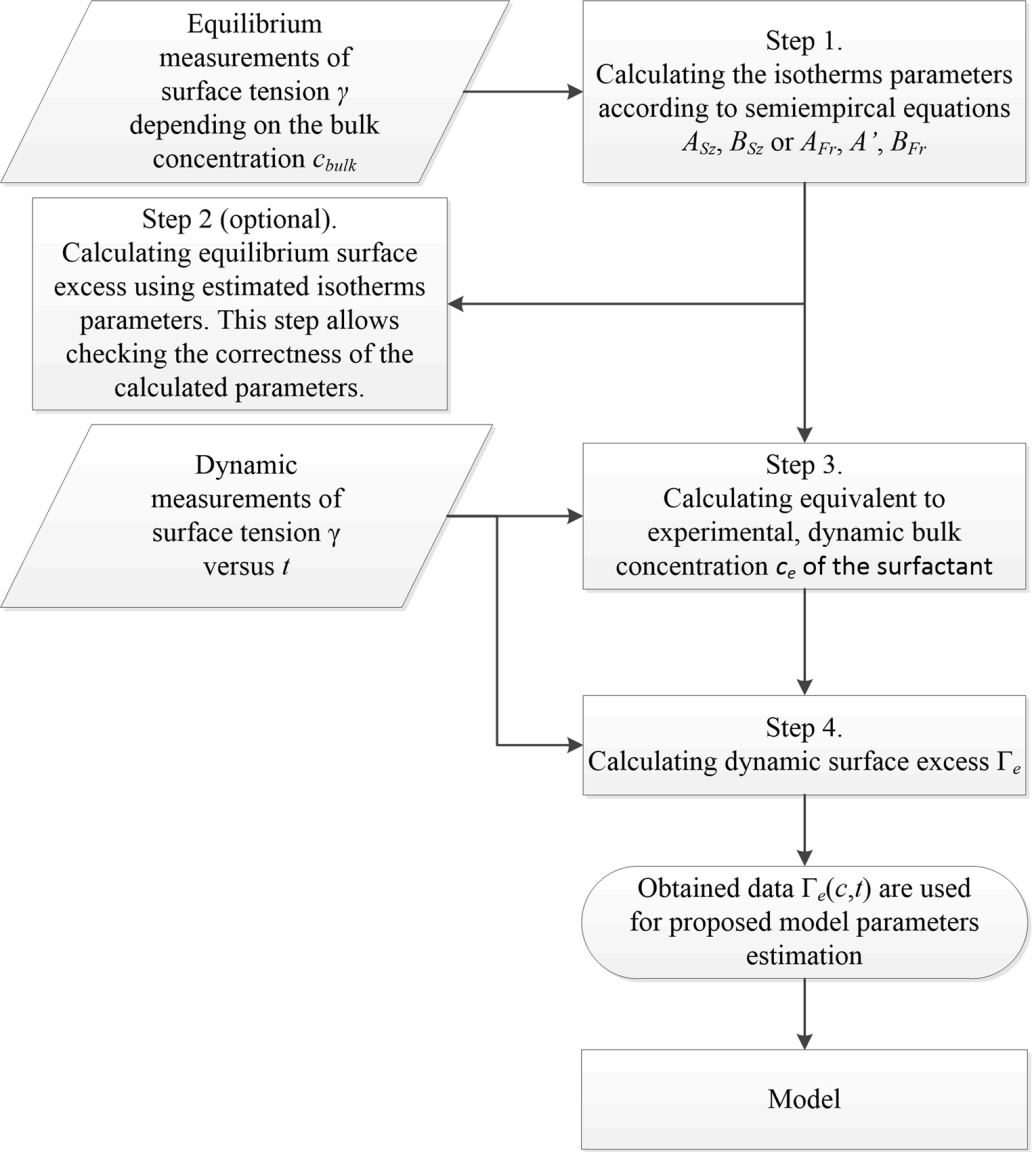
Sketch of modeling steps

Modeling of the experimental measurements requires estimation of the experimental (designated by subscript *e*) dynamic Gibbs surface excess values Γ_e_ and equivalent bulk phase concentrations *c*_*e*_. The procedure consists of several steps which are presented in Fig. 2. In the first step, the Szyszkowski or Frumkin isotherm parameters (*A*_Sz_, *B*_Sz_ or *A*_Fr_, *A*′, *B*_Fr_) are estimated because they are needed for further calculations. In the sense of the proposed method, this is an initial approximation of their values that will be improved in the next steps. The calculation is based on the equilibrium surface tension data *γ*(*c*_bulk_). Estimation of isotherms parameters is easily done by the typical least squares method which is used to adjust the fitting line to the experimental data. For that purpose several numerical tools can be used. The author used genfit from Mathcad [34] and also NonlinearFit from Maple [35] software. In most cases a relatively accurate estimation to the initial values is required. Tools exist for estimating the isotherm parameters [36] which simplifies the numerical minimization process under altering initial values for a given interval. This approach guarantees finding the global minimum of the objective function determined by minimization of the least squares error.

In the second step, which is in fact optional, when the isotherms parameters are given, the surface excess is estimated by Eqs. 28 and 33 respectively. The obtained Γ(*c*) values give important information on the correctness of estimated isotherms and the trend of changes of surface excess due to surfactant bulk concentration.

The third step is based on the assumption that for the transient state, when the adsorption process has not reached a steady state condition, the isotherm parameters can be used for further calculation. In fact these parameters describe the equilibrium state of the adsorption process and it is assumed that for specified transient state there is some equivalent equilibrium state. In the view of this statement, new equivalent parameters are introduced: the equivalent concentration of surfactant in the bulk phase *c*_e_ and corresponding equivalent surface excess Γ_e_. Those parameters are calculated on the basis of dynamic experimental measurements of surface tension *γ*_dyn_(*t*) by the use of the proper equations. In the case of modeling with Szyszkowski isotherm the equation reads:

37 $${\text{c}}_{\text{e}} \left( t \right){\text{ = exp}}\left( { - \frac{{\gamma_{\text{dyn}} \left( t \right) - \gamma_{0} }}{{\gamma_{0} B_{\text{Sz}} }}} \right)A_{\text{Sz}} - A_{\text{Sz}} ,$$

In the case of Frumkin isotherm the *c*_e_ calculation can only be done by the use of a numerical method because Eq. 31 cannot be solved analytically for concentration *c*. This issue is in fact straightforward and typical numerical methods for nonlinear problems can be used. The tool from Mathcad (root function or given/find block), Maple (fsolve procedure) or numerical libraries package IMSL (subroutine ZBREN [37]) can be used. These tools do not cover all the possibilities available, they are mentioned here because the author used them for the calculations for the method presented.

The estimated concentrations from the dynamic measurements are used in the fourth step to calculate the equivalent dynamic surface excess Γ_e_. For every time measurement the calculation is as follows:

- for Szyszkowski isotherm
  38 $$\Gamma _{e} (c_{e} ,t) = \frac{{B_{\text{Sz}} \gamma_{0} c_{e} (t)}}{{RT\left( {c_{e} (t) + A_{\text{Sz}} } \right)}},$$
- for Frumkin isotherm
  39 $$\Gamma _{e} (c_{e} ,t) = \frac{{\gamma_{0} c_{e} (t)}}{RT}\left( {\frac{{B_{\text{Fr}} }}{{c_{e} (t) + A_{\text{Fr}} }} + \frac{{2A_{\text{Fr}} A^{{\prime }} c_{e} (t)}}{{\left( {c_{e} (t) + A_{\text{Fr}} } \right)^{3} }}} \right).$$

In this way, based on experimental results, the calculated values of Γ_e_ versus time *t* and concentration *c*_e_ are obtained to be used in Eqs. 29 and 34 depending on isotherm used.

### Experimental Procedure

**Fig. 3:**
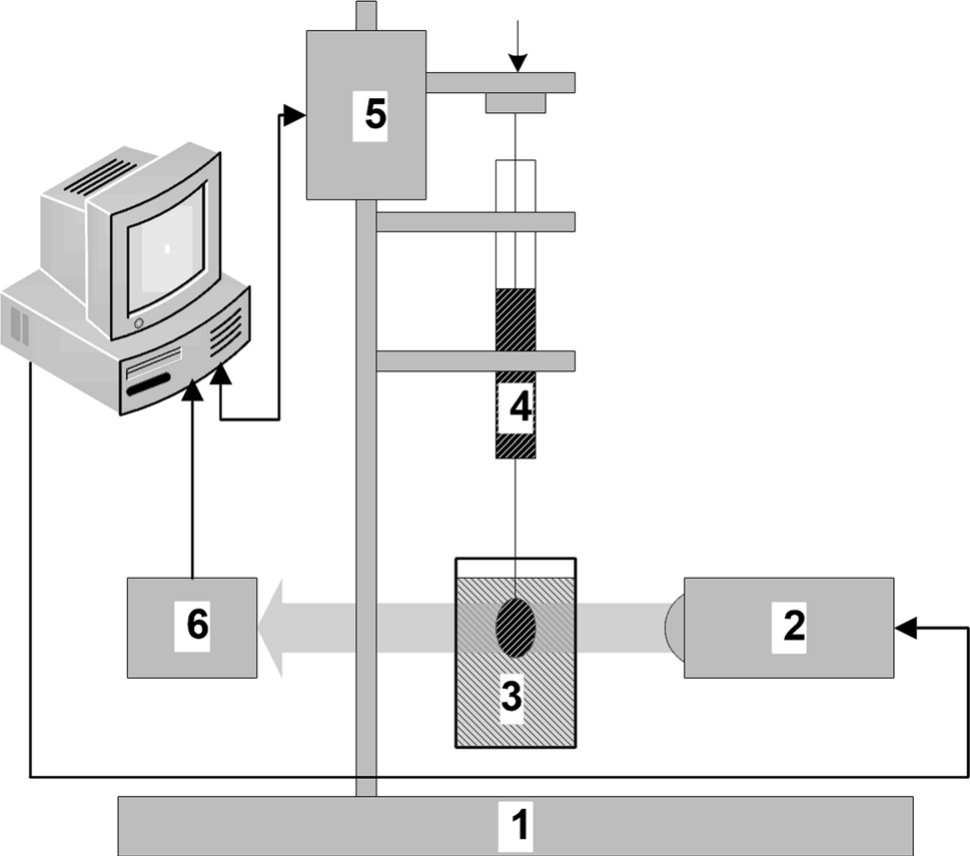
Tracker tensiometer assembly sketch

The experimental verification was done using the liquid/liquid system water and toluene. The experimental setup is shown in Fig. 3. A Tracker tensiometer (IT Concept-Teclis) was used for the drop shape measurements [38–41].

The software managing the measurement sequence controls the flow rate of liquid from the syringe 4 with a motor driven piston 5, recording at the same time the image obtained from the camera 6. Proper recording is possible by using a uniform light source 2. Removable basis 1 allows precise setting of the field of view and focus at the area of the recorded image of the drop shape. During the measurement, software records the volume of the liquid droplet and its shape. On the basis of the droplet size, the software calculates the surface tension, contact angle, the droplet radius, the droplet surface area and its volume. The camera performs measurements for a drop hanging from normal and inverted vertical capillary or for a droplet lying on a selected surface.

**Fig. 4:**
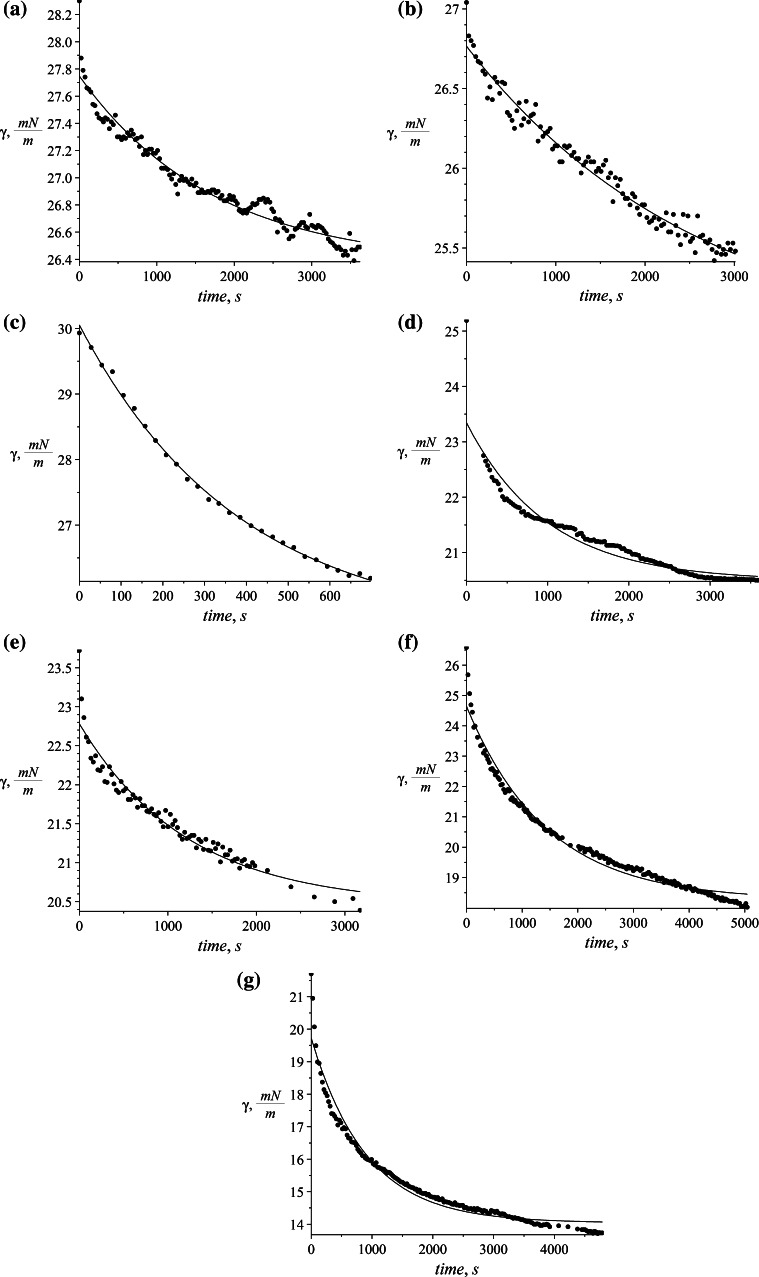
Changes of surface tension in time in different surfactant concentrations. **a** 0.035 mol/m^3^, **b** 0.052 mol/m^3^, **c** 0.104 mol/m^3^, **d** 0.347 mol/m^3^, **e** 0.520 mol/m^3^, **f** 0.520 mol/m^3^, **g** 5.201 mol/m^3^

In order to prepare two phase mixtures, aqueous solutions of appropriate SDS concentration were shaken with toluene for 4 h to achieve a state of saturation while maintaining equal volumes of the phases. The flasks were allowed to stand for phase separation for 24 h. Several equilibrium measurements with altered surfactant concentration were done which are necessary to determine the adsorption isotherms. Also the dynamic measurements of surface tension changes in time were performed. Injecting the SDS solution droplet from the capillary creates a fresh interface between the saturated phases. The subsequent observation of transient changes in the shape of a droplet gives a picture of changes in interfacial tension, and conducting the measurement until the changes are very small, determines the equilibrium of adsorption. It is assumed that when the changes are less than 10^−4^ N/m the system reached the equilibrium state [42]. The changes in dynamic surface tension at different surfactant concentrations given in Table 1 are shown in Fig. 4.

**Table 1:** Concentrations of SDS used in the experimental work

Sample	1	2	3	4	5	6	7
SDS concentration, g/l (mol/m^3^)	0.01 (0.035)	0.015 (0.052)	0.03 (0.104)	0.1 (0.347)	0.15 (0.52)	0.3 (1.04)	1.5 (5.201)

The equilibrium state for the investigated system was determined for SDS concentrations that do not exceed the critical micelle concentration which equals 2.26 g/L [43]. The equilibrium surface tension *γ*_∞_ and parameters of chosen isotherms using Eqs. (31) and (33) were estimated based on the obtained resultant measurements. The equilibrium values of surface tension were calculated by least squares fit to Eq. 40.

40 $$\gamma = a \cdot \exp (b \cdot t) + c,$$

Using the values of *a*, *b* and *c* parameters, the equilibrium surface tension is calculated in the limit when time *t* approaches infinity using Eq. 41.

41 $$\gamma_{\infty } = \mathop {\lim }\limits_{t \to \infty } a \cdot \exp (b \cdot t) + c.$$

Equation 40 is arbitrarily assumed by proper choice of nonlinear interfacial tension changes. Therefore the *a*, *b* and *c* parameters are only of statistical significance and do not represent any physical quantity. To calculate their values two different tools were chosen: Mathcad using the genfit procedure and Maple using the NonlinearFit procedure. Both gave different result but the difference is not relevant. The residual values (estimated fit error) for both tools are less than 10^−6^ (8.71 × 10^−7^ and 6.72 × 10^−7^ respectively). Maple software was used for the model calculation due to its analytical capabilities. The parameter values are presented in Table 2. The quality of the fit is expressed by the *R*^2^ determination parameter.

**Table 2:** Fitting parameters from Eq. 38

Sample	1	2	3	4	5	6	7
*a*	1.43	2.15	4.56	2.60	2.36	6.27	5.47
*b*	−4.99 × 10^−4^	−3.06 × 10^−4^	−2.69 × 10^−3^	−5.67 × 10^−4^	−6.36 × 10^−4^	−6.55 × 10^−4^	−1.03 × 10^−3^
*c*	26.27	24.58	25.48	20.11	20.25	18.18	13.99
*R* ^2^	0.96	0.97	0.99	0.98	0.97	0.98	0.97

**Fig. 5:**
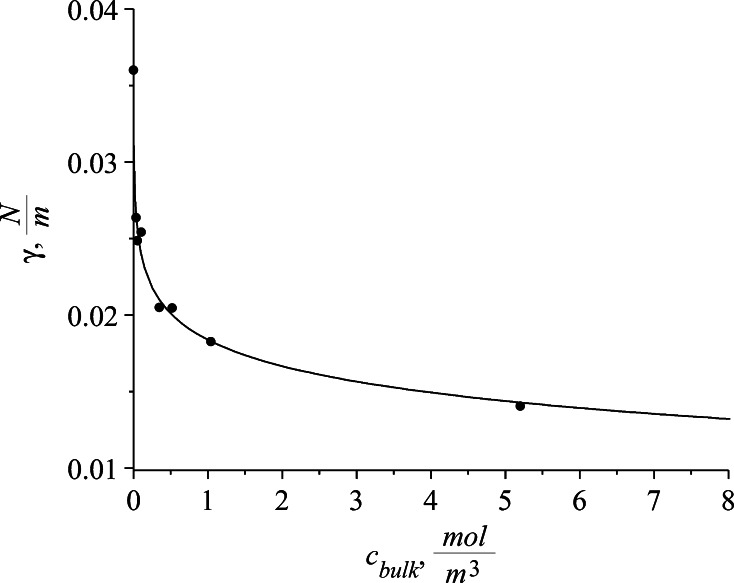
Equilibrium surface tensions in different bulk concentrations of SDS. The presented Szyszkowski and Frumkin isotherms are presented by a *single line*

**Fig. 6:**
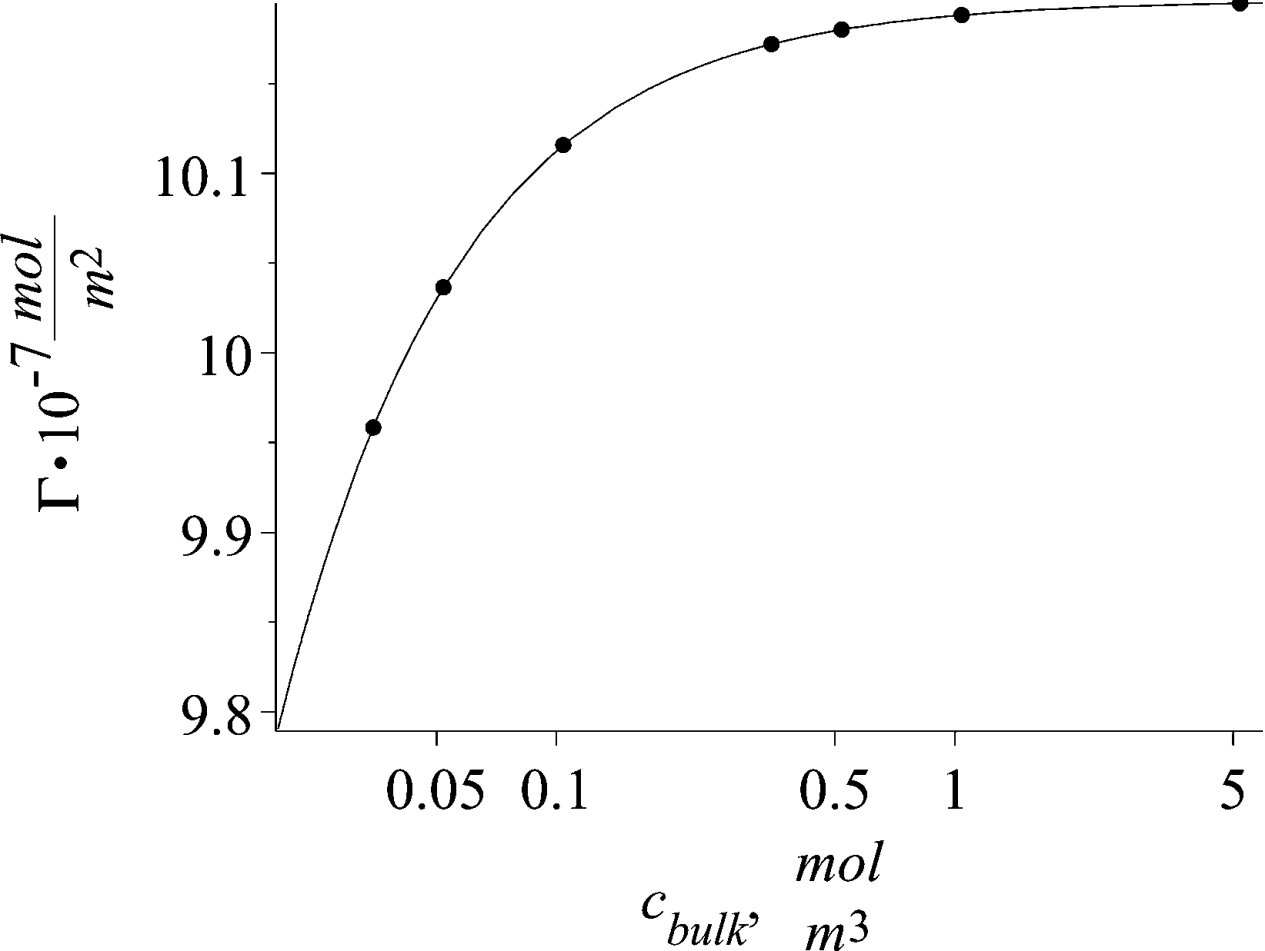
Szyszkowski and Frumkin isotherms in toluene/water solution of SDS

The parameter values of the best fit isotherm model are given in Table 3. The quality of the fit is expressed by the residual sum of squares (RSS) for both the isotherms. It is also visible by examining the RSS values that due to additional parameter the fit to the Frumkin isotherm is a little better but the difference is small. Based on the estimated isotherms parameters the surface tension and surface excess as a function of concentration are presented in Figs. 5 and 6. The fit to both of the isotherms is almost identical so the solid line represents both the Szyszkowski and Frumkin isotherms.

**Table 3:** Calculated isotherm parameters

Parameter	Szyszkowski isotherm	Frumkin isotherm
*A* (mol/m^3^)	8.29 × 10^−4^	5.03 × 10^−4^
*B*	6.90 × 10^−2^	6.91 × 10^−2^
*A*′	N/A	−3.52 × 10^−2^
RSS	3.36 × 10^−6^	3.35 × 10^−6^

The *A* and *B* coefficients correspond to the Szyszkowski (*A*_Sz_, *B*_Sz_) and Frumkin (*A*_Fr_, *B*_Fr_) isotherms respectively

### Calculations

**Fig. 7:**
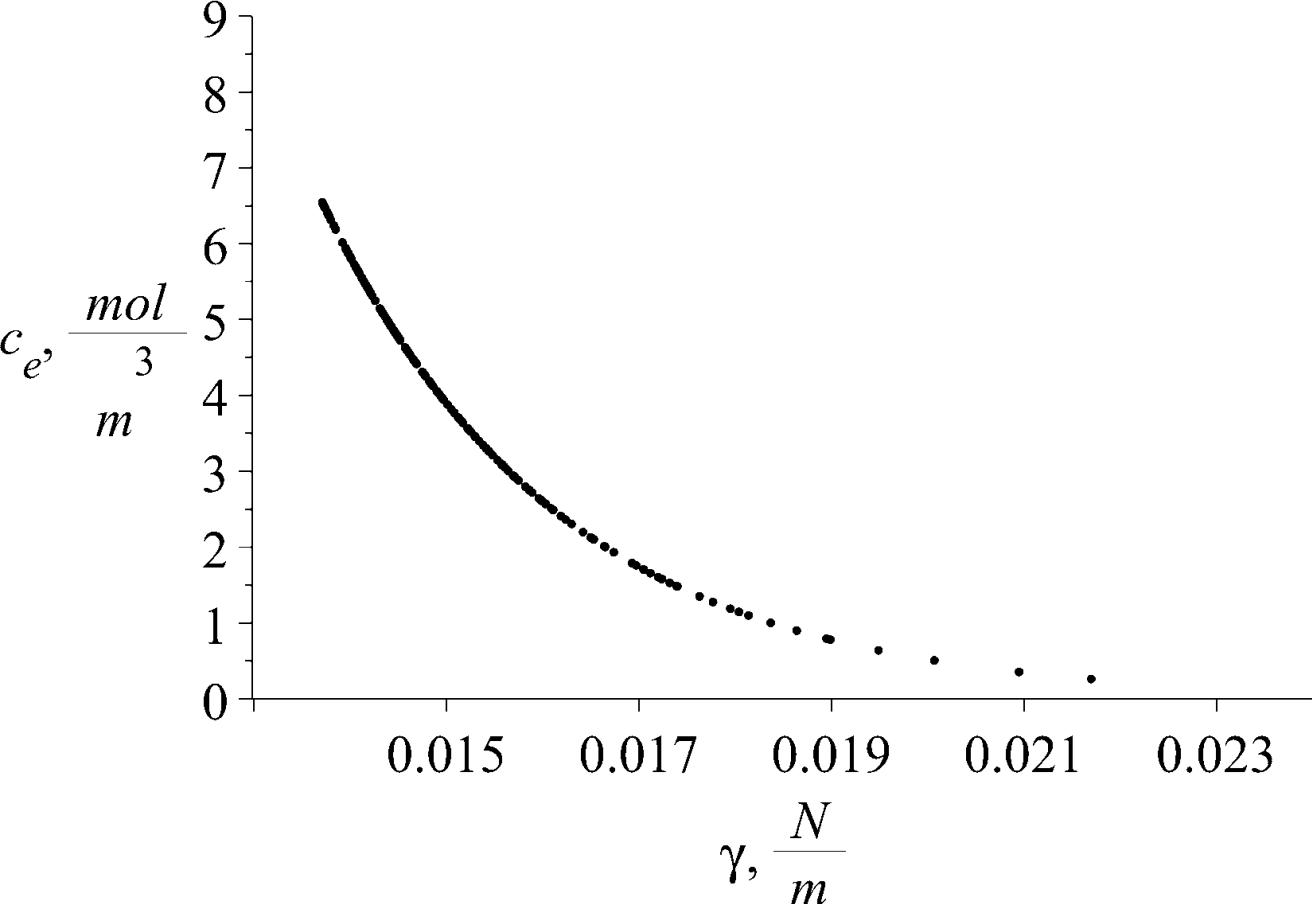
Typical profile of concentration equivalent to the corresponding surface tension *c*_e_ = *f*(*γ*) estimated for SDS concentration equal to 5.201 mol/m^3^. The *dashed line* denotes the CMC of SDS at 8.3 mol/m^3^

Having estimated the isotherms parameters, the equivalent surfactant concentration *c*_e_ is obtained using Eq. 36 for the Szyszkowski isotherm and the numerical method when the Frumkin isotherm is used. The equivalent concentration *c*_e_ as a function of interfacial tension for a given sample set is presented on Fig. 7. The profile shows the tendency to increase the equivalent concentration with decreasing interfacial tension. However the extrapolated values do not reach nonphysical concentration above the CMC for SDS.

The next step is to estimate the equivalent dynamic surface excess Γ_e_ for the equilibrium state. The model assumes that the isotherms are used to estimate the transient states of surface excess approaching equilibrium. Determination of the parameters for the proposed model including diffusion coefficient in the adsorption sublayer, the thickness of this layer, and numerical fine-tuning of the isotherm parameters is realized by fitting the model function to the experimental data. The model parameters were calculated using the minimization procedure LSSolve contained in the NAG [44] numerical library. The minimization procedure is used to obtain best fit of the proposed model to the experimental data. That is done by reducing the error which is expressed by the sum of squares of the model deviations from the equivalent surface excess Γ_e_ for a given time *t* and equivalent bulk concentration *c*_e_.

The minimization problem is stated as follows:

42 $$\hbox{min} \left( {\left( {\Gamma _{e} (c_{e} ,t) -\Gamma (c_{e} ,t)} \right)^{2} } \right)$$

where Γ_e_(*c*_e_, *t*) is the experimental equivalent surface excess and Γ(*c*_e_, *t*) is the model function given by Eq. 29 or 34 depending on the chosen isotherm. The problem is solved using the least squares method subject to the following variable boundary conditions:

- *ω* > 0
- *D*_ads_ > 0
- *L* > 0

The bounds on the diffusion coefficient *D*_ads_ and size *L* are reasonable physiochemically. The *ω* parameter bound results from the mathematical structure of the model and is applied to obtain only real value solutions.

## Results

**Fig. 8:**
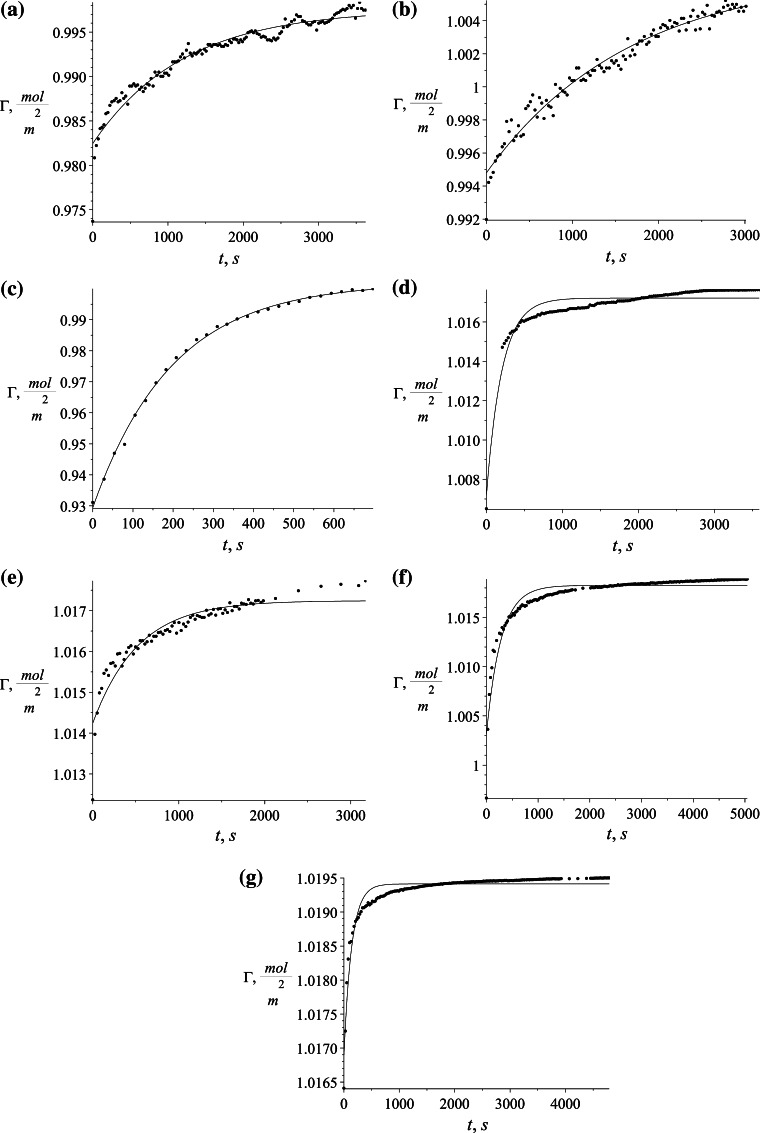
Values of Γ_e_ = *f*(*t*) × 10^−6^ using the Szyszkowski isotherm for several different SDS concentrations. **a** 0.035 mol/m^3^, **b** 0.052 mol/m^3^, **c** 0.104 mol/m^3^, **d** 0.347 mol/m^3^, **e** 0.520 mol/m^3^, **f** 0.520 mol/m^3^, **g** 5.201 mol/m^3^

**Fig. 9:**
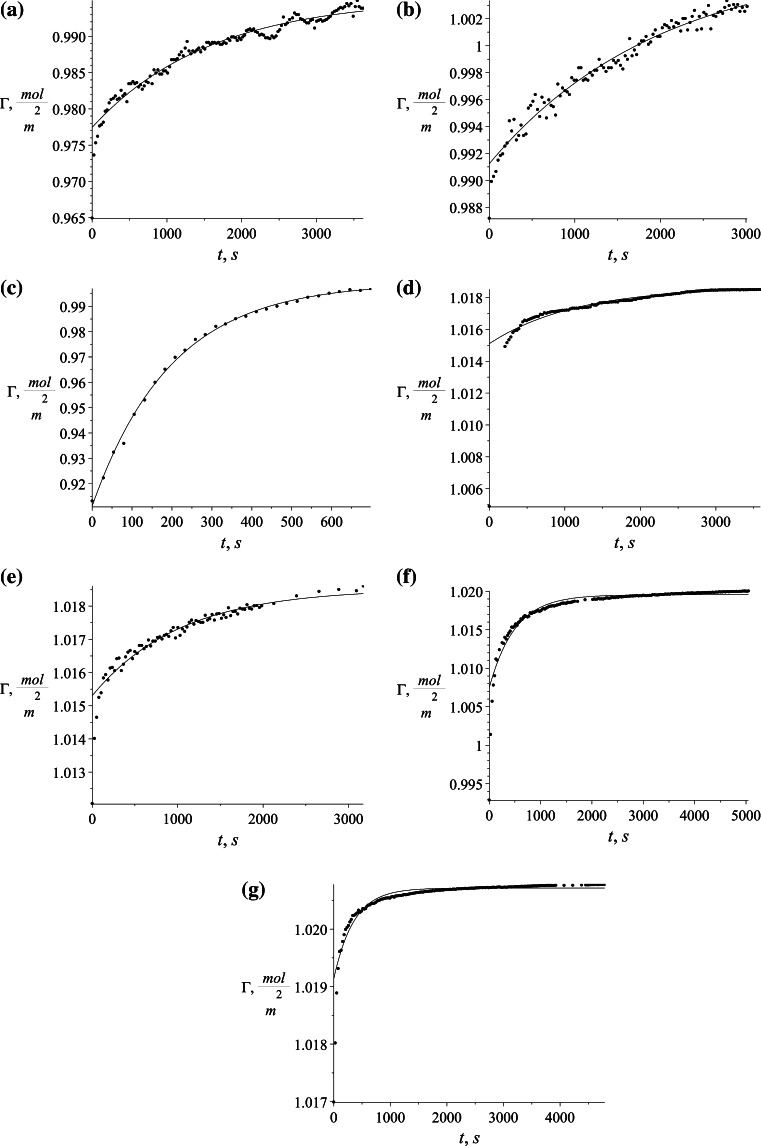
Values of Γ_e_ = *f*(*t*) × 10^−6^ using the Frumkin isotherm for several different SDS concentrations. **a** 0.035 mol/m^3^, **b** 0.052 mol/m^3^, **c** 0.104 mol/m^3^, **d** 0.347 mol/m^3^, **e** 0.520 mol/m^3^, **f** 0.520 mol/m^3^, **g** 5.201 mol/m^3^

The proposed model can be used with any isotherm relating surface excess with bulk surfactant concentration. In our case, two different isotherms were chosen and the solutions obtained are presented below. For both isotherms, the first step is to calculate the discrete set of points Γ_e_ corresponding to the experimental values of surface excess. They are calculated for Szyszkowski isotherm using Eq. (38) and for Frumkin isotherm using Eq. (39). The values of Γ_e_ refer to measured surface tension *γ* during process time *t*. The calculated values of Γ_e_ for Szyszkowski and Frumkin isotherm respectively are presented in Figs. 8 and 9 on succeeding graphs (a–g) for the specified SDS concentrations (see Table 1).

The shape of time evolution of surface excess is almost identical for both isotherms used. The difference is visible in their magnitude where the values calculated using Szyszkowski isotherm are higher by about half a percent. The calculated values (Figs. 8, 9) are approximated by the empirical equation:

43 $$\Gamma = s_{1} \cdot \exp (s_{2} \; \cdot \;t) + s_{3} ,$$

and was chosen to fit the character of the data and also to be able easily to define the derivative located in the boundary condition (8).

### Model with Szyszkowski Isotherm

The values obtained in previous step are used for the calculation using the model proposed by Eq. (29). In the case of the two-parameter Szyszkowski isotherm (28) the minimization problem (42) becomes a seven-parameter problem. Following are the parameters calculated by fitting the model to the experimentally derived values of Γ_e_:

- *a*, *b*—Johannsen equation parameters (21),
- *A*_Sz_, *B*_Sz_—Szyszkowski isotherm (28),
- *D*_ads_—diffusion coefficient (6),
- *L*—size of adsorptive sublayer (7),
- *ω*—integration parameter (11).

The best fit of the model Eq. (29) to the experimental surface excess Γ_e_ is achieved for the values presented in Table 4. The Szyszkowski isotherm parameters are convergent with the values estimated by typical method in described earlier (see Step 1 at Fig. 2). The additional kinetic parameters *D*_ads_ and *L* obtained realistic values. The effective diffusion coefficient magnitude of 10^−9^ m^2^/s is a typical value found for similar surface active substances [45–48].

**Table 4:** The result of constrained minimization of the model given by Eq. 29

Parameter	*A*_Sz_ (mol/m^3^)	*B*_Sz_ (–)	*D*_ads_ (m^2^/s)	*L* (m)	*a* (–)	*b* (–)	*C*_0_ (1/m^2^)
	9.29 × 10^−4^	6.89 × 10^−2^	9.84 × 10^−9^	4.75 × 10^−7^	7.45 × 10^3^	3.05 × 10^2^	9.67 × 10^6^

**Fig. 10:**
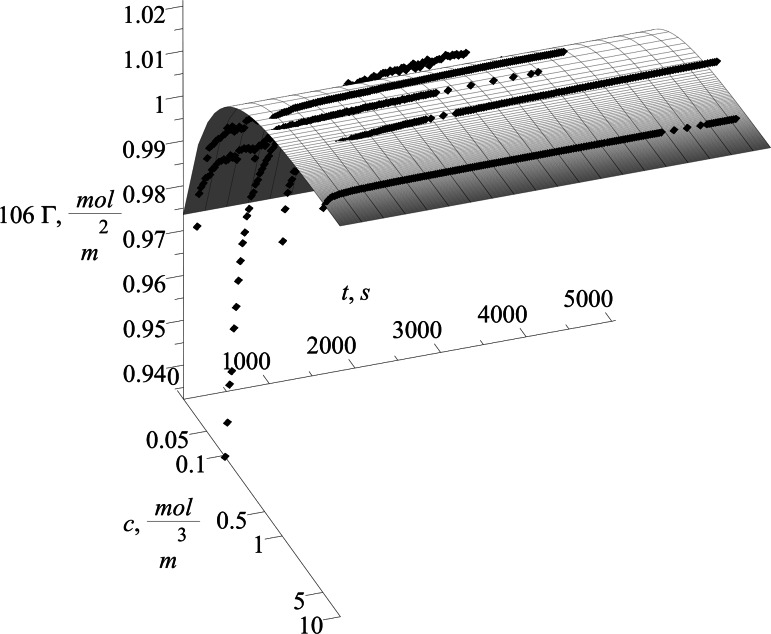
The model using the Szyszkowski isotherm presented as the surface together with the experimental points *filled diamonds*

The quality of the fit of the model is presented on the Fig. 10. It is visible that for a longer time the model fits data much better than for a short time. It is explained by the fact that the model utilizes isotherms as one step of calculation, which in principle are defined for equilibrium states that is longer process time.

**Fig. 11:**
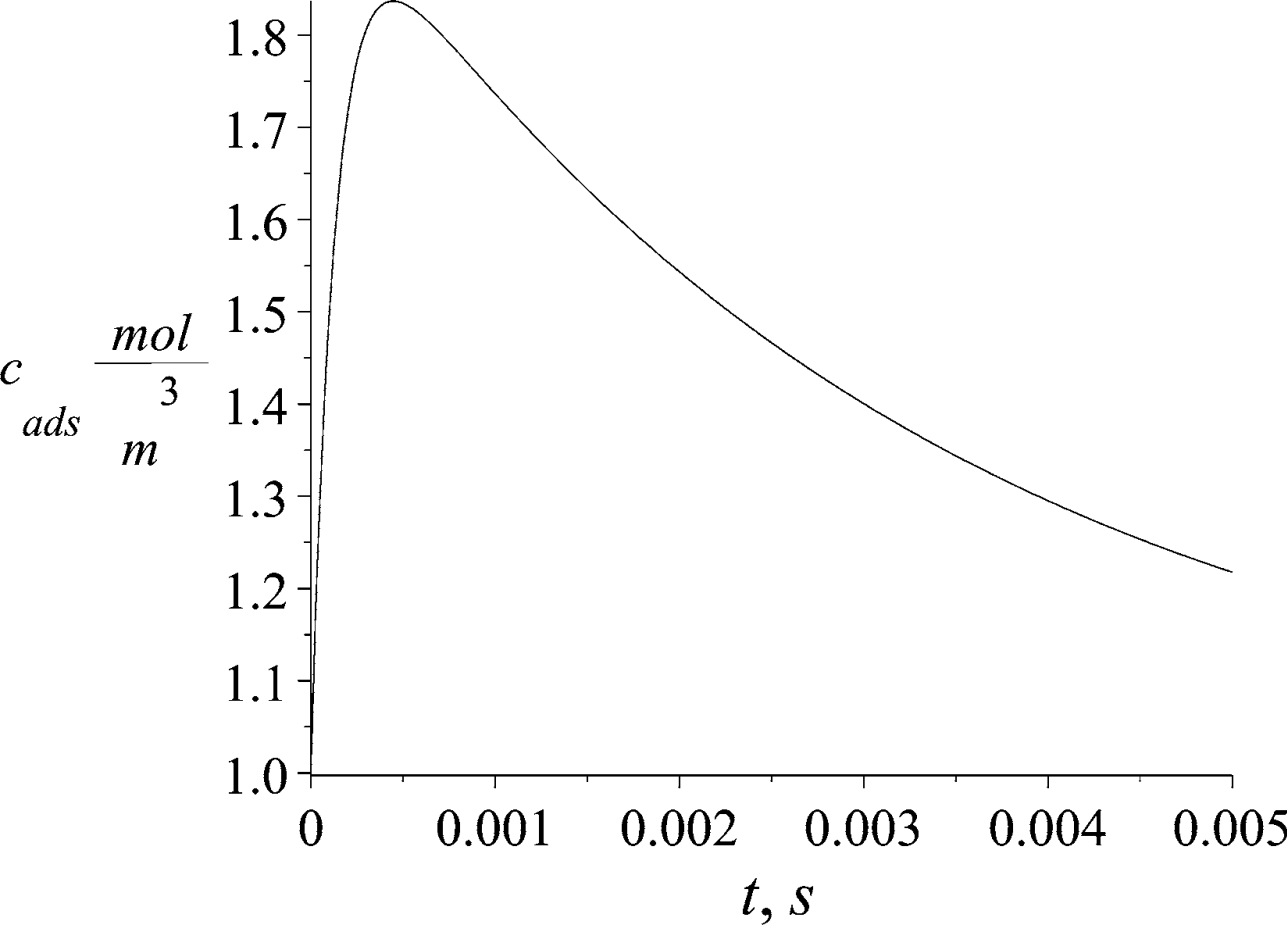
SDS concentration dynamics at the interface by estimated Johannsen profile

The calculated parameters of Johannsen Eq. (21) *a* and *b* describe the dynamics of the surfactant *c*_ads_ concentration in the adsorptive sublayer (Fig. 11). Visible and characteristic maximum refers to very short process time that is not detectable by typical tensiometer equipment. Such a maximum, if its existence might be experimentally proved, can be explained by initially fast surfactant transfer into the sublayer and then with slower adsorption to the interface.

### Model with the Frumkin Isotherm

The model proposed using the Frumkin isotherm is given by Eq. 34. The calculations are similar to those for the Szyszkowski isotherm model so they are not repeated. The focus in the text is on the comparison between both approaches. The first difference appears in the Γ_e_ values. The Frumkin isotherm is a three-parameter equation that results in an eight-parameter minimization solution to Eq. 42. The parameters which are estimated by model using this approach are:

- *a*, *b*—Johannsen equation parameters (21),
- *A*_Fr_, *B*_Fr_, *A*′—Frumkin isotherm parameters (33),
- *D*_ads_—diffusion coefficient (6),
- *L*—size of adsorptive sublayer (7),
- *ω*—integration parameter (11).

The estimated parameters for the Frumkin isotherm model are presented in Table 5. Comparing the *A* parameter which has direct impact on the subsequently computed value of surface excess, it has about 8 % lower value than for the Szyszkowski isotherm, while the *B* parameter is equal for both isotherms. The negative value of *A*′ indicates a presence of attractive interactions existing in the adsorptive sublayer. An approximate two percent difference between calculated values of diffusion coefficients and less than one percent difference for the size of the adsorptive sublayer are not relevant. The Johannsen Eq. (21) parameters and *ω* values are also almost identical. This indicates that both model approaches, using two distinct isotherms, are convergent and stable regardless of isotherm chosen.

**Table 5:** The results of constrained minimization of Eq. 34

Parameter	*A*_Fr_ (mol/m^3^)	*B*_Fr_ (–)	*A*′ (–)	*D*_ads_ (m^2^/s)	*L* (m)	*a* (–)	*b* (–)	*C*_0_ (1/m^2^)
	8.52 × 10^−4^	6.90 × 10^−2^	−1.19·10^−2^	9.68 × 10^−9^	4.78 × 10^−7^	7.45 × 10^3^	3.08 × 10^3^	9.67 × 10^6^

### Adsorptive Sublayer Description

The results obtained allow us to draw a description of the adsorptive sublayer on the model basis. The effective diffusion coefficient *D*_ads_ and size of the sublayer *L* are used to build a concentration profile in this region. The retyped Eq. (6) that describes the mass transfer due to the adsorption process reads:

44 $$D_{\text{ads}} \cdot \frac{{\partial^{2} }}{{\partial x^{2} }}c_{\text{ads}} \left( {x,t} \right) = \frac{\partial }{\partial t}c_{\text{ads}} \left( {x,t} \right)$$

The Eq. (44) is solved using boundary condition proposed:

45 $$\begin{aligned} x &={} 0,\quad D_{\text{ads}} \frac{\partial }{\partial x}c_{\text{ads}} \left( {0,t} \right) = \frac{{{\text{d}}\varGamma }}{{{\text{d}}t}}, \hfill \\ x &={} L,\quad c_{\text{ads}} \left( {L,t} \right) = c_{\text{bulk}} . \hfill \\ \end{aligned}$$

and with initial condition:

46 $$c_{\text{ads}} \left( {x,0} \right) = \left\{ {\begin{array}{*{20}c} 0 & {x < L} \\ {c_{\text{bulk}} } & {x \ge L} \\ \end{array} } \right..$$

**Fig. 12:**
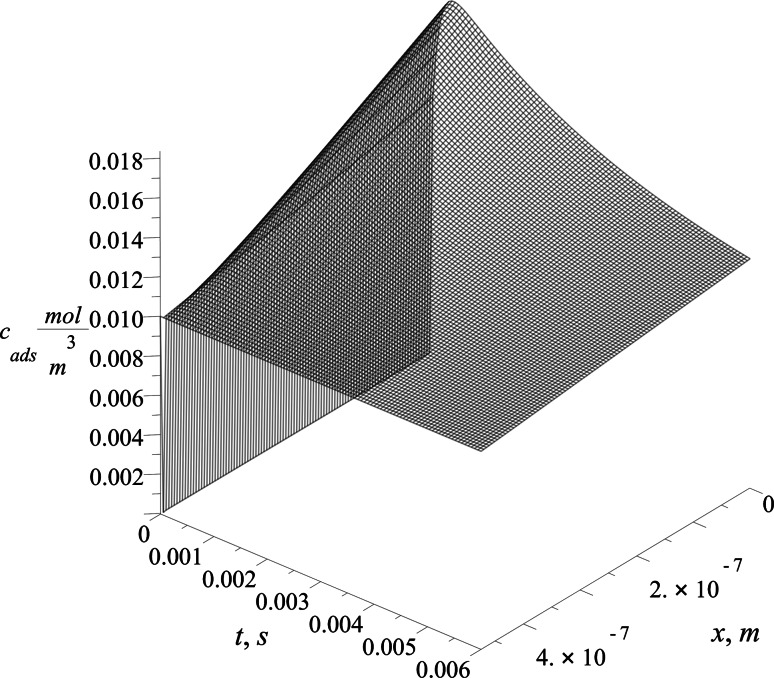
SDS concentration profile in the adsorptive sublayer solved using values from the Szyszkowski isotherm

The boundary condition at *x* = 0 applies that surfactant flux to the interface is equal to the change of the surface excess. The right-hand side of this condition is formulated using Eq. (22). The boundary condition at *x* = *L* describes surfactant transport with constant bulk concentration of surfactant. The initial condition applies no presence of surface active substance in adsorptive sublayer at time *t* = 0. This assumes that fresh interface is present at the beginning of the adsorption process. The solution to the initial-boundary problem stated is presented on the Fig. 12.

The concentration profile shown presents the dynamics of evolution of the surfactant in the adsorptive sublayer in the (*x*, *t*) system. In the initial stage of the process, for a very short time, the sudden change in the character of the profile is revealed. The typical, exponentially decaying diffusion profile changes into a profile dominated by the adsorption at the very initial stage of the process. The character of the concentration profile becomes linear for a longer time of the adsorption. The flux of the surfactant to the interface in terms of diffusion law is estimated by the Eq. (22). The differences between two approaches using two isotherms are best compared in the solution space (*x*, *t*). In order to obtain such a result the relative difference Δ was calculated:

47 $$\Delta \left( {x,t} \right) = \frac{{c_{{{\text{ads}}_{\text{Fr}} }} \left( {x,t} \right) - c_{{{\text{ads}}_{\text{Sz}} }} \left( {x,t} \right)}}{{c_{{{\text{ads}}_{\text{Fr}} }} \left( {x,t} \right)}}.$$

**Fig. 13:**
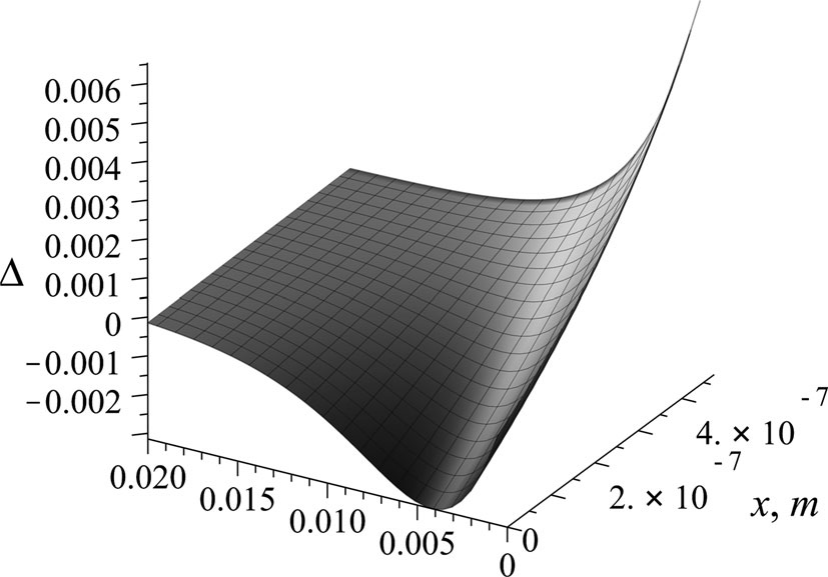
Relative differences in the adsorptive sublayer concentration solution obtained by solving the Szyszkowski and Frumkin isotherm models

This difference in profile is given in Fig. 13 using adsorptive sublayer concentrations that resulted from the two models solutions, using Szyszkowski and Frumkin isotherms.

The differences between both approaches are small and reach their maximum at about six percent of the relative value for *x* = *L* (bulk phase boundary) and initial time *t* = 0. The difference is not significant due to the very short time and is presented to accent its existence. For longer time values the difference approaches zero.

The last result that is useful for computation in different fields of simulation is the surfactant interface mass flux. During the process of developing the model one of the steps is the formulation of the mass flux of the surfactant to the interface. The formulation is given by Eq. (22). The mass flux of component *i* is defined by the Eq. (48):

48 $$J_{i}^{\text{dyf}} = \frac{{{\text{d}}\Gamma _{i} }}{{{\text{d}}t}}$$

**Fig. 14:**
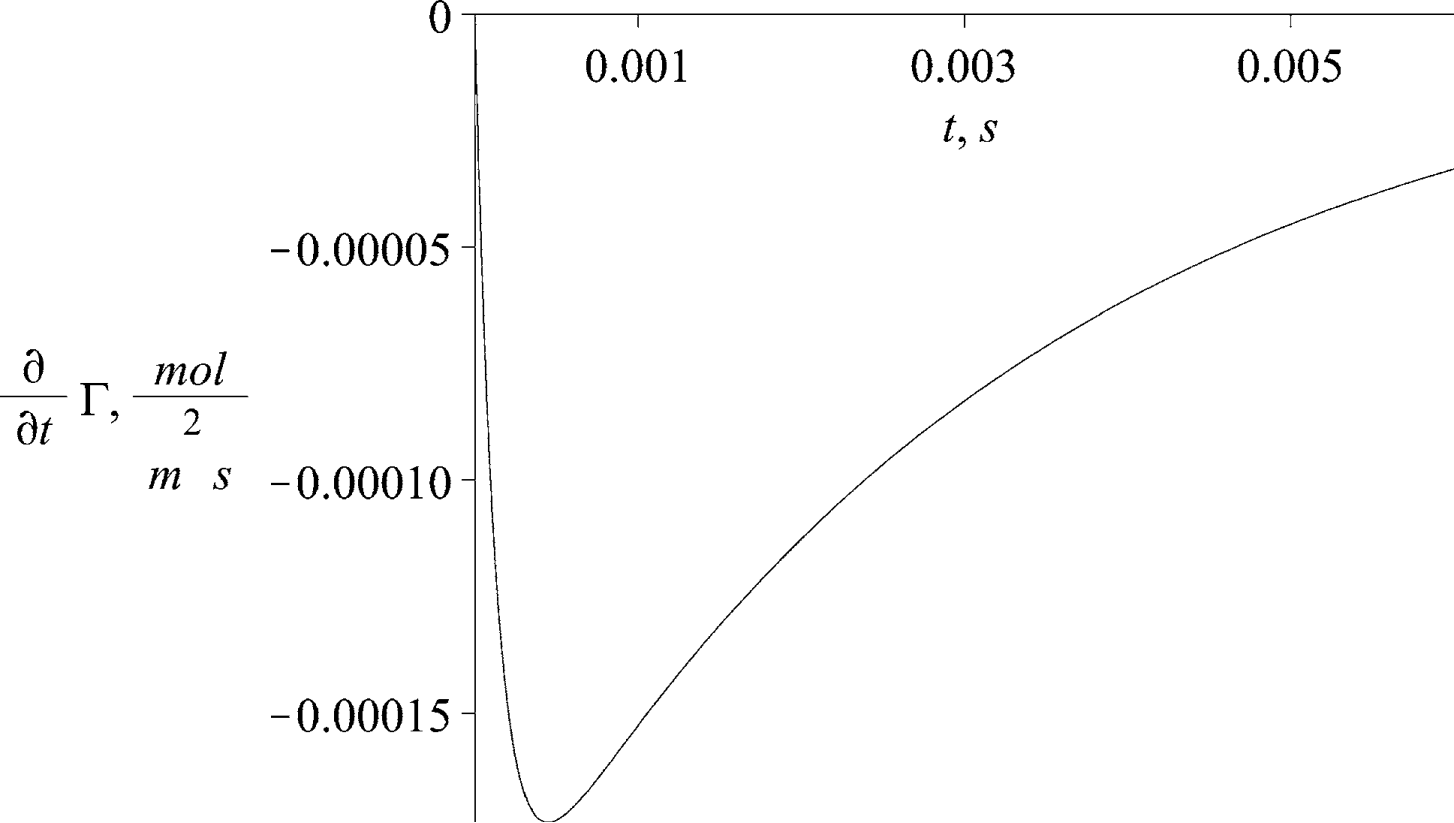
The time profile of adsorptive mass flux of the surfactant

The obtained time profile of adsorptive mass flux gives negative values due to the formulation of the coordinate system in which the location at *x* = 0 presents the interface. Consequently the flux is formulated as a mass diffusive flux from the bulk phase to the interface. The flux reaches a steady value for long times at about −2.24 × 10^−8^ mol/m^2^ s as shown in Fig. 14.

## Conclusions

The work presented includes the mathematical model of the adsorption process at the interface. The model was developed in two distinct variants using Szyszkowski and Frumkin isotherms. The model was developed based on the Fick’s second law for transient diffusion. To apply this approach to the adsorptive sublayer where the concentration gradient is not the primary driving force for mass transfer, the effective diffusion coefficient was introduced. Such an approach is widely used in the literature [1–5] and also by the author [49–51] for distinct cases.

The specific subspace, called the adsorptive sublayer, was distinguished and characterized by the model equation with boundary and initial conditions applied. It is assumed that surface excess is altered by the mass transfer of the surfactant from the sublayer to the interface. The mass transfer itself in the sublayer is dependent also on the bulk surfactant concentration. The model was solved by an analytical method to formulate the solution in the most general way.

The model was verified using experimental results for the water solution of SDS with the organic phase as toluene. The estimated diffusion coefficients are of the order 10^−9^ m^2^/s and correspond to the literature data. The calculated size of the sublayer is of the order 10^−7^ m. From the mathematical assumption applied and analogy to the boundary layer theory such size is considered to be reasonable.
